# Editorial: Flexibility in the genome and proteome: an adaptive toolkit for organisms

**DOI:** 10.3389/fgene.2023.1229315

**Published:** 2023-07-07

**Authors:** Tarun Jairaj Narwani, Gaurav Sharma, Alexandre G. de Brevern

**Affiliations:** ^1^ Institut National de la Santé et de la Recherche Médicale (INSERM), Paris, France; ^2^ Université Paris Cité and Université de la Réunion and Université des Antilles, INSERM, BIGR, DSIMB, Paris, France; ^3^ Department of Biotechnology, Indian Institute of Technology Hyderabad, Sangāreddi, Telangana, India

**Keywords:** flexibility, protein disorder, retrogenesis, evolution, protein protein interaction (PPI), COVID-19, protein complex analysis

At the dawn of this millennium, the Human Genome Project (HGP) prospected the most detailed description of the genetic and epigenetic regulatory pathways (The International Human Genome Mapping Consortium et al., 2001). However, it focussed on covering the euchromatic fraction while neglecting the heterochromatic regions which would eventually turn out to be of crucial importance. It would not be until 2 decades later that an almost complete human reference genome is published (Nurk et al., 2022). The telomere-to-telomere length reference genome, T2T-CHM13, covers the existing gaps and presents a wholistic sequence for the potential evolutionary relics to be uncovered in due course of time. During the decadal time gap between the first reference genome and the latest, numerous genome-wide studies have been published and have recognised linked associations (Mosaku et al., 2023). The validation of the robustness of their results lies with the time hereafter.

Such milestones have proven to be catalytic in advancing the technological barriers. An exciting example could be the invention of molecular 3D structure prediction methods like, AlphaFold (DeepMind, 2023) and RoseTTaFold (Baek et al., 2021) which use deep learning neural networks to access repetitive patterns from the protein sequence space that can be associated to the known 3D structural space to learn the relationship between the two. Building upon these advancements, a collaborative effort led by Deepmind and EMBL-EBI have used AlphaFold to predict the structural space of the known human proteome (Tunyasuvunakool et al., 2021). However, the structural space that was available to train these networks lack important regions, particularly disordered and highly flexible regions. This could be the reason for these method’s inability to explain the low confidence of structure prediction in the known flexible regions or the impact of pathological mutations on the structure.

Scientific milestones usually result in attracting research groups who would design their work-plan around that exciting discovery. Although quite natural, it often leads to a very pertinent question that does the designed project consider the complex nature of biological systems by assessing the inherent flexibility of the molecular system being studied. The aforementioned examples demonstrate the caveats of neglecting the inherent flexibility of the genomes and proteomes. This flexibility makes the biological systems complex to study and understand. Therefore, it is important that we assert a similar flexibility in our approaches to broaden our understanding of life.

This Research Topic was therefore aimed at compiling original research that could impart flexibility to our understanding of complex biomolecular systems. For example, two groups studied protein disorders in two very different systems and concluded the role of inherent flexibility towards their adaptive advantage. Chandra et al. investigated the disordered region of the antitoxin from the toxin-antitoxin (TA) pair in the pathogen *M. tuberculosis*. This bacterium has nine MazEF family toxin-antitoxin systems, which have a significant impact on the persistence and survival of *Mycobacterium tuberculosis* within their host. Using charged residue (Asp) scanning mutagenesis at the intrinsically disordered regions, they showed a TA interaction module unique to MazEF6 system. They established that N-terminus of the antitoxin (MazE) that is usually expected to be involved in DNA binding could bind to the toxin (MazF) instead. These counter-expected results highlight that structure prediction built on evolution may not work for predicting correct binding mode in MazEF6 TA pair. Thus, pointing out the specificity of individual antitoxins towards their partner toxins that is likely to provide selective evolutionary advantage to the organism.

Chauhan and Sowdhamini performed an in-depth analysis of LIM domains of the Cysteine and Glycine-rich Protein 3 (CSRP3), a key protein implicated in dilated and hypertrophic cardiomyopathies. CSRP3 is structurally characterized by two LIM domains (each containing two Zinc fingers) connected by a disordered region. Zinc fingers are well characterised as DNA binding domains and are highly conserved. However, comparing (>5,000) sequences of LIM domain across the tree of life, authors observe a trimodal distribution for their length variations which they attributed to the varying length of the disordered linker. Using MD simulation experiments, they have asserted the role of the disordered region in facilitating the LIM domain interactions which can vary among different species. An interesting finding from the phylogeny of CSRP3 was the identification of a homolog in freshwater microspecies which are not known to have a circulatory network, thus, scratching a curious gateway into the origins of CSRP3 gene.

Yu and collaborators (Yu et al.) are interested in understanding the function of X-derived retrogenes that are known to contribute towards genetic diversity during evolution. These genes are usually expressed in the testes and perform important functions during spermatogenesis. Phylogenetic analysis showed that a retrogene and an X-linked maintenance gene are subject to purifying selection and hence may have evolutionarily conserved functions. However, they have no functional role in spermatogenesis as shown by mice experiments. This study has provided direct evidence that some autosomal X-derived retrogenes may be non-functional in spermatogenesis, thus, challenging our understanding that flexibility imparts adaptive advantage.

Arangasamy et al. have performed the most extensive analysis of the SF3b Spliceosomal complex to date. They analysed the molecular signatures that contribute to sequence divergence and functional specialization of SF3b from 578 eukaryotic species. They showed that the location and formation of subcomplexes can influence the conservation of individual protein components. For example, proteins located at the surface of the SF3b complex show more sequence variation than those located at the core. Although the biological implication of such divergence is not immediately clear, it can confer adaptive features in individual components of the multi-protein SF3b complexes and may contribute to its functional adaptability.

Finally, a brief research report by Sultana and collaborators (Sultana et al.) establishes two important biomarkers, the C-reactive protein (CRP) and D-dimer in 228 Bangladeshi COVID-19 patients. They observed a significant association between the severity of COVID-19 and the two biomarkers within the population. Such studies are aimed at making the diagnostics precise by considering ethnic populations and their varied immune responses against a disease.

Overall, this research topic explores the role of flexibility by studying various systems that range from retrogenes to multi-protein complexes to disordered systems while providing insight into each system and their selective advantage imparted by their inherent flexibility.
